# Stress Degradation Behavior of Atorvastatin Calcium and Development of a Suitable Stability-Indicating LC Method for the Determination of Atorvastatin, its Related Impurities, and its Degradation Products

**DOI:** 10.3797/scipharm.1208-06

**Published:** 2012-10-09

**Authors:** Pallavi Vukkum, J. Moses Babu, R. Muralikrishna

**Affiliations:** 1 Analytical Research, Custom Pharmaceutical Services, Dr. Reddy’s Laboratories Ltd., Bollaram road, Miyapur, Hyderabad-500049 (AP), India.; 2 Department of Chemistry, Andhra University, Visakhapatnam-530003, India.

**Keywords:** Atorvastatin calcium, RP-HPLC, Stability-indicating method, Forced degradation, LC-MS, Validation

## Abstract

A rapid, reversed-phase liquid chromatographic method was developed for the quantitative determination of Atorvastatin calcium, its related substances (12 impurities), and degradation impurities in bulk drugs. The chromatographic separation was achieved on a Zorbax Bonus-RP column by employing a gradient elution with water–acetonitrile–trifluoroacetic acid as the mobile phase in a shorter run time of 25 min. The flow rate was 1.0 mL/min and the detection wavelength was 245 nm. The drug substance was subjected to stress studies such as hydrolysis, oxidation, photolysis, and thermal degradation, and considerable degradation was observed in acidic hydrolysis, oxidative, thermal, and photolytic stress conditions. The formed degradation products were reported and were well-resolved from the Atorvastatin and its related substances. The stressed samples were quantified against a qualified reference standard and the mass balance was found to be close to 99.5% (w/w) when the response of the degradant was considered to be equal to the analyte (i.e. Atorvastatin), which demonstrates the stability-indicating capability of the method. The method was validated in agreement with ICH requirements. The method developed here was single and shorter (25 min method for the determination of all 12 related impurities of Atorvastatin and its degradation products), with clearly better resolution and higher sensitivity than the European (85 min method for the determination of six impurities) and United States pharmacopeia (115 min and 55 min, two different methods for the determination of six related substances).

## Introduction

Atorvastatin calcium, chemically (3*R*,5*R*)-7-[2-(4-Fluorophenyl)-5-isopropyl-3-phenyl-4-(phenylcarbamoyl)-1*H*-pyrrol-1-yl]-3,5-dihydroxyheptanoic acid calcium salt (2:1), is an inhibitor of the 3-hydroxy-3-methylglutaryl-coenzyme A (HMG-CoA) reductase. This enzyme catalyzes the conversion of HMG-CoA to mevalonate, an early and rate-limiting step in cholesterol biosynthesis. Atorvastatin is administered as the calcium salt of the active hydroxyl acid and between 10 and 80 mg per day is used to reduce the raised lipid levels in patients with primary hyperlipidemia (familial and non-familial) or combined hyperlipidemia [1–3].

Several procedures have been reported in the literature such as LC/MS/MS [4–7], microbore LC/ESI-MS/MS [8], LC with electrospray tandem mass spectrometry [9, 10], the micellar electrokinetic capillary chromatographic method [11], spectrophotometric methods [12], and LC methods with UV detector [13–17] for the determination of Atorvastatin in biological samples, aqueous samples, and tablets. To the best of our knowledge, no single rapid stability-indicating LC method was reported for the determination of Atorvastatin calcium, its 12 potential impurities, and degradants in the bulk drugs and in pharmaceutical dosage forms. The pharmacopeia methods were much longer in run time: the European pharmacopeia method was 85 min for the determination of only six impurities, and the United States pharmacopeia mentions two different methods in 115 min and 55 min for the determination of the six related substances.

The objective of this research work was to develop a stability-indicating method with a shorter run time (with 1/4 of the run time reported) for the determination of twelve related substances, degradation products, and assay of Atorvastatin calcium in a single run. Forced degradation studies were performed on the drug substance to show the stability-indicating capability of the method. All of these studies were performed in accordance with established ICH guidelines. The mixture of the degraded samples and their related substances were used to optimize the method. The method was also validated as per ICH requirements.

## Experimental

### Compounds

Samples of Atorvastatin calcium and its potential impurities were synthesized by the process research department of Dr. Reddy’s Laboratories, Hyderabad, India. HPLC grade Acetonitrile, analytical grade sodium hydroxide, hydrochloric acid, and hydrogen peroxide were purchased from Rankem (Mumbai, India). Trifluoroacetic acid was purchased from Across Organics (Geel, Belgium). High purity water was prepared using a Millipore Milli-Q Plus (Millipore, Milford, MA, USA) purification system.

### Instrumentation

Using an Alliance Waters 2695 separations module LC system with diode array detector, the method development attempts were made, and subsequently the method validation and forced degradation were done. A reverse phase Zorbax Bonus-RP column (150 X 4.6 mm and 3.5 μm as particle size) (Agilent, USA) was used for achieving the separation of all compounds. The chromatographic data were recorded using an HP-Vectra (Hewlett Packard, Waldron, Germany) computer system with millennium data acquisition software. LC-MS was performed using the Agilent 1100 series liquid chromatography system coupled with a 6410-series triple quadruple mass spectrometer (Palo Alto, CA, USA). A Cintex digital water bath was used for the hydrolysis studies. The photostability study was carried out in a photostability chamber (Sanyo, Leicestershire, UK). The thermal stability study was carried out in a dry air oven (Cintex, Mumbai, India).

### Chromatographic conditions

The chromatographic separation was achieved on Zorbax Bonus-RP column (150 × 4.6 mm and 3.5 μm as particle size). The gradient LC method employed water: trifluoroacetic acid in the ratio of 100:0.10 (v/v) as mobile phase A and acetonitrile: trifluoroacetic acid in the ratio of 100:0.10 (v/v) as mobile phase B. The flow rate of the mobile phase was 1.0 mL/min. The LC gradient program was set as (T/ %B) = 0/40, 10/50, 15/70, 20/90, and 25/90 with a post run-time of 5 min. The column temperature was maintained at 40 °C and the detection wavelength was set at 245 nm. The column loading was optimized as 5 μg of Atorvastatin calcium in a 10 μL injection volume. A mixture of water and acetonitrile in an equal ratio was used as a diluent.

### LC–MS conditions

The LC–MS system (Agilent 1100 series liquid chromatography system coupled with a 6410 series triple quadruple mass spectrometer) was used for the identification of unknown compounds formed during forced degradation and for peak homogeneity confirmation. The same LC conditions were directly employed for LC-MS. The analysis was performed in both positive and negative electro spray ionization modes. The capillary voltage was 4.0 kV. The source temperature was 300°C and the gas flow rate was 600 L/h. The main advantage of this method is the ability to directly employ the same LC conditions to LC-MS.

### Preparation of standard solutions and sample solutions

A solution of Atorvastatin calcium was prepared at a concentration of 500 μg/mL in the diluent for the related substances and assay determination. The stock solution of each impurity was prepared at a concentration of 500 μg/mL in the diluent. The standard solutions were prepared by diluting the stock solutions to attain the required concentration of impurities and drug substances with the diluent. A stock solution with the mixture of all 12 potential impurities was also prepared and a consequent quantity was spiked with Atorvastatin at a level of 0.15% w/w of the target analyte concentration (TAC), which was 500 μg/mL.

### Stress studies/specificity

Specificity is the ability to assess the analyte unequivocally in the presence of its potential impurities [18] which may be expected to be present like the other impurities, degradants, matrix, etc. [19]. The specificity of the developed LC method for Atorvastatin calcium was established in the presence of its potential impurities, namely Imp-A, Imp-B, Imp-C, Imp-D, Imp-F, Imp-G, Imp-H, Imp-I, Imp-J, Imp-K, Imp-L, Imp-M (Imp-E was 3S, 5S enantiomer of Atorvastatin, so it was not captured in this method), and its degradation products. The ability of the method to separate all of the compounds was assessed by evaluating the resolution between the peaks corresponding to the various compounds to show the stability-indicating ability and specificity of the proposed rapid LC method.

**Fig. 1:**
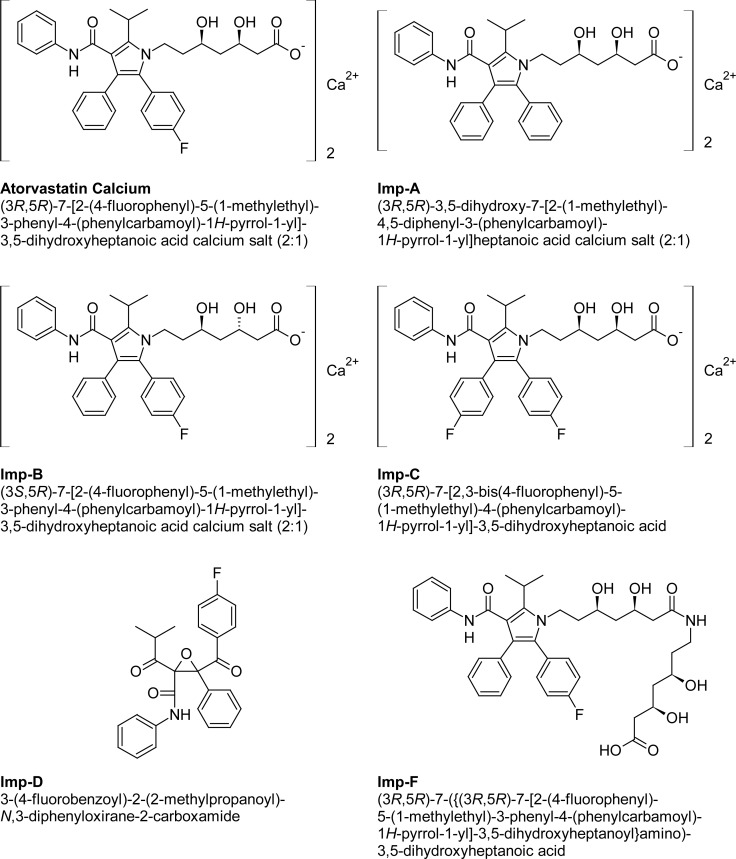

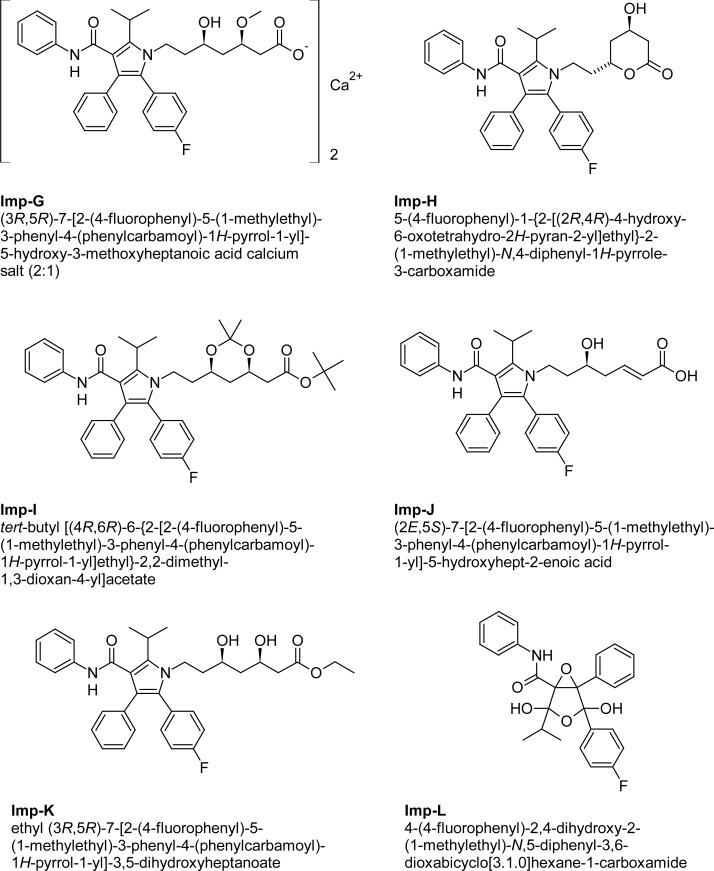

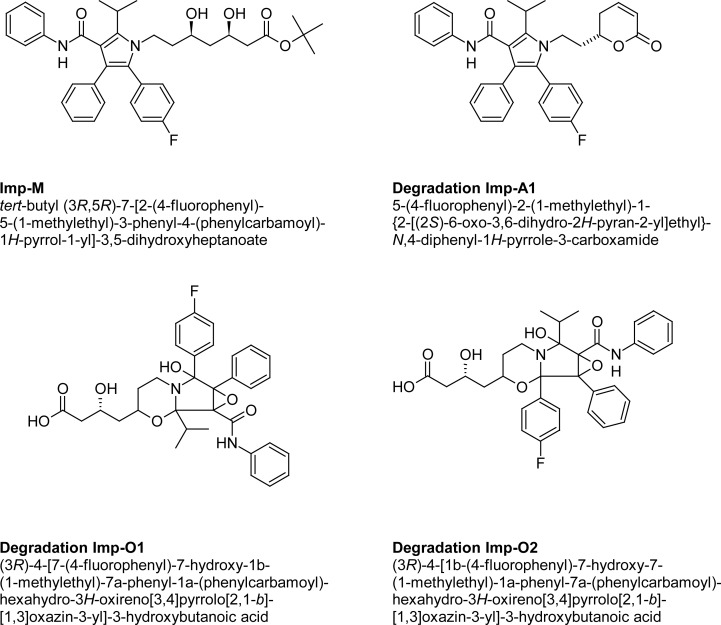
Structures and names of Atorvastatin calcium, its related impurities, and its forced degradation impurities

The stress conditions employed for degradation studies as per ICH recommendation include photolytic, thermal, oxidation, and hydrolysis with acid and base. The photolytic stress study was performed for 11 days at 200 W h/m^2^ of UV light and 1.2 million lux hours of visible light. The thermal stress study was performed at 105°C for 10 days. The acid, base stress studies were performed with 0.1 N HCI for 24 h and 1 N NaOH for 42 h at ambient temperature (25 ± 2°C). The oxidation stress was done with 1% H2O2 solution for 24 h at ambient temperature (25 ± 2°C). All of the stressed samples were quantified against the Atorvastatin calcium reference standard. The peak purity of Atorvastatin in the stressed samples and the spiked solution of Atorvastatin with its known related impurities were checked by the photodiode array detector (PDA), and also the mass numbers of the unknown degradation impurities were checked by LC-MS. The structures of Atorvastatin calcium, its related impurities, and degradation products are shown in Fig. 1.

## Method validation

The described LC method has been extensively validated for the assay and related substances as per ICH guidelines [20].

### System suitability test

The system suitability test is an integral part of chromatographic methods and is used to verify that the resolution and reproducibility of the chromatographic system are adequate for the analysis to be performed. The system suitability test results of the developed LC method on the Zorbax Bonus-RP column are computed in Table 1.

### Limit of detection (LOD) and limit of quantification (LOQ)

The LOD and LOQ of Atorvastatin calcium and its 12 potential impurities were determined by diluting their stock solutions to known concentration solutions that would yield a signal-to-noise ratio of 3:1 and 10:1 respectively. Precision was carried out at the LOQ level by preparing six individual preparations of Atorvastatin with its related impurities at the LOQ level, and by calculating the percentage RSD for the areas of Atorvastatin and its related impurities. Accuracy at the LOQ level was also carried out by preparing three recovery solutions of Atorvastatin with its related impurities at the LOQ level, and by calculating the percentage recovery for areas of all related impurities.

### Linearity

The linearity of the method was established at two different levels. The assay linearity was performed by preparing five different solutions of Atorvastatin from 80 to 120% w/w (80, 90, 100, 110, and 120% w/w) with respect to TAC. The linearity at a low level was performed by preparing six different solutions from the LOQ to 0.30% w/w (LOQ, 0.05, 0.10, 0.15, 0.20, and 0.30% w/w) of Imp-A, Imp-B, Imp-C, Imp-D, Imp-F, Imp-G, Imp-H, Imp-I, Imp-J, Imp-K, Imp-L, Imp-M, and Atorvastatin calcium with respect to TAC. The peak area versus concentration data was plotted for linear regression analysis. The correlation coefficients of regression, slope, intercept, and percent y-intercept of the calibration curves were computed. The relative response factor (RRF) of each impurity was determined by dividing the slope of each impurity by the slope of Atorvastatin calcium.

### Precision

Six individual measures of Atorvastatin calcium were performed with 0.15 % w/w of each of the 12 potential impurities to the reference of TAC. The assay and the content of each impurity were determined for each of the preparations and the method precision was evaluated by calculating the percentage RSD of assay values and each of the impurity’s content in six preparations. Experiments with a different analyst, column, and instrument in the same laboratory were performed in order to ascertain the intermediate precision or ruggedness of the developed method.

### Accuracy

The accuracy of the assay was evaluated in triplicate at three concentration levels, i.e. 400, 500, and 600 μg/ mL of Atorvastatin calcium. The percentage recovery at each level was calculated. Standard addition and recovery experiments were conducted to determine the accuracy of the related substances in the same method for the quantification of all 12 impurities in Atorvastatin calcium. The study was carried out in triplicate at 0.075, 0.15, and 0.225% w/w of the target analyte concentration. The percentage recoveries for each of the 12 impurities were calculated by considering the amount of impurity spiked, amount of impurity available in an un-spiked sample, and amount of impurity recovered after RRF correction.

### Robustness

The robustness of an analytical procedure is a measure of its capacity to remain unaffected by small, but deliberate variations in method parameters and provides an indication of its reliability during normal usage. To determine the robustness of the developed LC method, deliberate changes were made from original experimental conditions. The flow rate of the mobile phase was 1.0 mL/min. To study the effect of flow rate on the theoretical plates, tailing, and resolution, it was changed to 0.8 and 1.2 mL/min. The effect of wavelength was studied at 243 nm and 247 nm, instead of at 245 nm. The effect of column temperature was studied at 35 and 45 °C instead of 40 °C. The effect of the ratio of the organic modifier was studied by varying ±2% (absolute) of both mobile phase-A and B from its original condition. For all changed conditions, i.e. flow rate, wave length, temperature, and organic modifier, the system suitability results were computed like theoretical plates, tailing factor of analyte, and the resolution between the analyte, Imp-B, and Imp-C.

### Solution stability and mobile phase stability

The solution stability of Atorvastatin calcium in the presence of its related impurities at the specification level was carried out by keeping the solution in a tightly capped volumetric flask at room temperature (25±2 °C) on a laboratory benchtop and at refrigerator conditions (5±2 °C) for 48 h. The assay of the analyte and content of all potential impurities were determined at 4 h intervals up to the study period 48 h.

The mobile phase stability was carried out by evaluating the assay of the analyte and content of all related impurities in the Atorvastatin calcium sample solution which was spiked with known impurities at the specification level, the spiked solution was prepared freshly at each 4 h interval up to 48 h and injected, while the same mobile phase was used during the study period.

## Results and discussion

### Method development and optimization

**Fig. 2:**
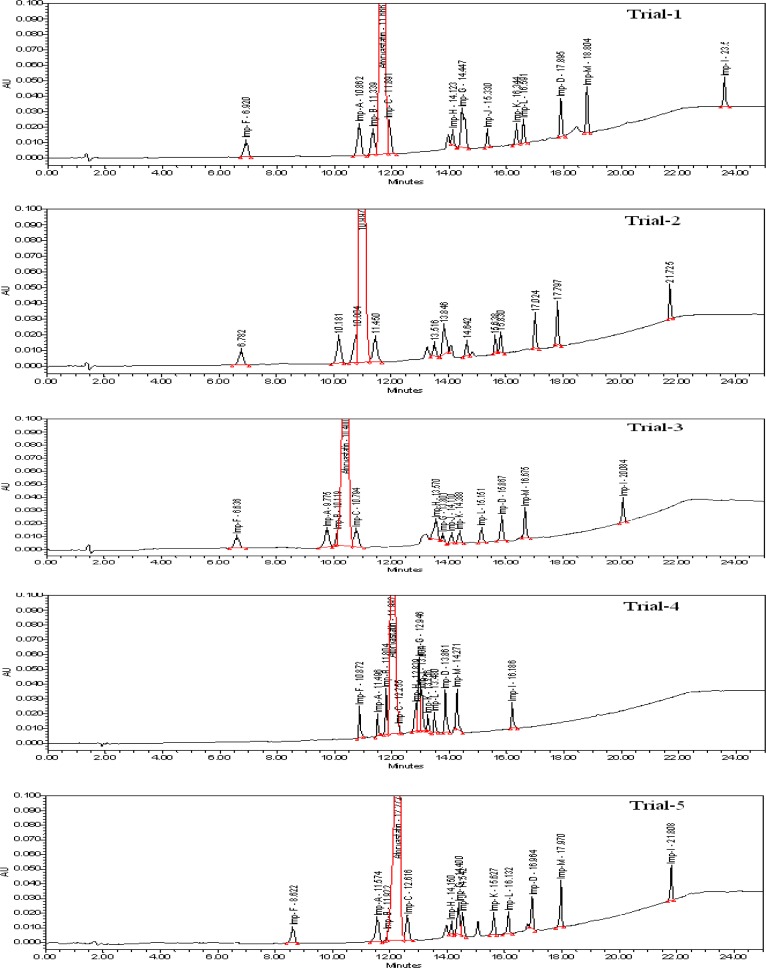
Typical chromatograms of method development

**Fig. 3:**
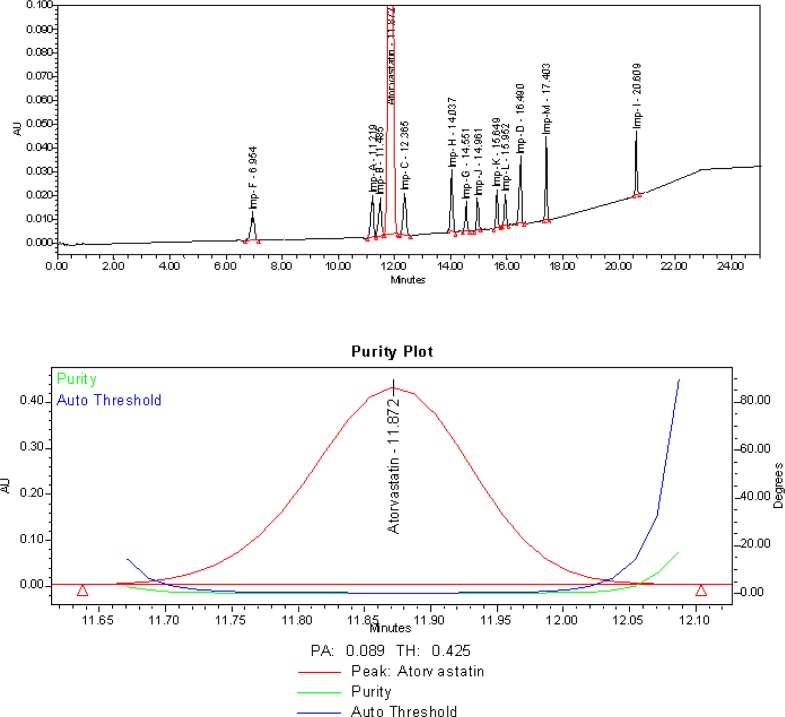
Typical HPLC chromatogram and peak purity spectrum of Atorvastatin calcium spiked with its related impurities at specification level

Imp-A, Imp-B, ImpC, Imp-D, Imp-F, Imp-G, Imp-H, Imp-I, Imp-J, Imp-K, Imp-L, and Imp-M (Imp-E was 3S, 5S enantiomer of Atorvastatin, so it was not captured in this method and the Imp-D will undergo a transformation equilibrium with its cyclic hemiketal form, which is nothing but the Imp-L) are the potential impurities of the Atorvastatin calcium drug substance. The core objective of the chromatographic method is to get the better peak shape of the Atorvastatin and to separate all potential impurities and degradation products from the analyte, especially the critical impurities B & C. Considering the fact that the pKa value of Atorvastatin is 11.8 and is highly basic, it was focused to do the method development attempts in an acidic mobile phase. Trifluoroacetic acid is one of the excellent acidic additives which can change the selectivity with respect to the strength in the mobile phase compared to the other acidic additives and modifiers. The method developed for most of the recent weakly basic drugs involves trifluoroacetic acid to get an excellent peak shape. Initial attempts for the method development were made using a variety of stationary phases which are listed in Table 2 and Fig. 2. The impurities B & C are co-eluted with the analyte during the method development attempts on different stationary phases like C18, C8, cyano, and phenyl with different selectivity using water/acetonitrile/trifluoroacetic acid and a phosphate buffer as the mobile phase. The stationary phase has played a significant role in achieving the tailing factor of Atorvastatin and the separation between Atorvastatin, Imp-B, and Imp-C. The satisfactory peak shape and resolution of the closely eluting impurities were achieved on a Zorbax Bonus-RP column (150 X 4.6 mm and 3.5 μm particle size), by using solutions A and B as the mobile phase. The selected stationary phase is triple end-capped which is suitable especially for the basic compounds. The embedded, highly polar amide group of the stationary phase assists in deactivating unwanted silicon interactions, while propriety end-capping procedures complete the process to deactivate the chromatographic surface. The usage of the Zorbax Bonus-RP column is becoming popular for the analysis of basic compounds. When compared to the other stationary phases which are resulting in adverse peak shapes for the basic compounds, the Zorbax Bonus-RP has less residual metals, is durable at a lower pH, has a high quality of end-capping, and is suitable for our analyte. Solution A is a mixture of water: trifluoroacetic acid [100:0.10 (v/v)] and solution B is a mixture of acetonitrile: trifluoroacetic acid [100:0.10 (v/v)]. The flow rate of the mobile phase was 1.0 mL/min. The gradient program also played a vital role in the resolution of Imp-B & C with the Atorvastatin peak. The LC gradient program was optimized as (T/ %B) = 0/40, 10/50, 15/70, 20/90, and 25/90 with a post run-time of 5 min in order to get the better resolution between Imp-B, Atorvastatin, and Imp-C. At 40 °C column temperature, the retention time of the Atorvastatin calcium with the optimized gradient program was appropriate and the tailing factor of Atorvastatin was found to be around one. In the optimized conditions, it was observed that Atorvastatin calcium and its 12 potential impurities were well-separated with a resolution greater than two. The typical chromatogram of Atorvastatin calcium with its related impurities is presented in Fig. 3. The method was specific for Atorvastatin calcium from its potential impurities and degradant impurities. The system suitability test results are shown in Table 1.

## Results from Method validation

### Results of forced degradation studies

**Fig. 4:**
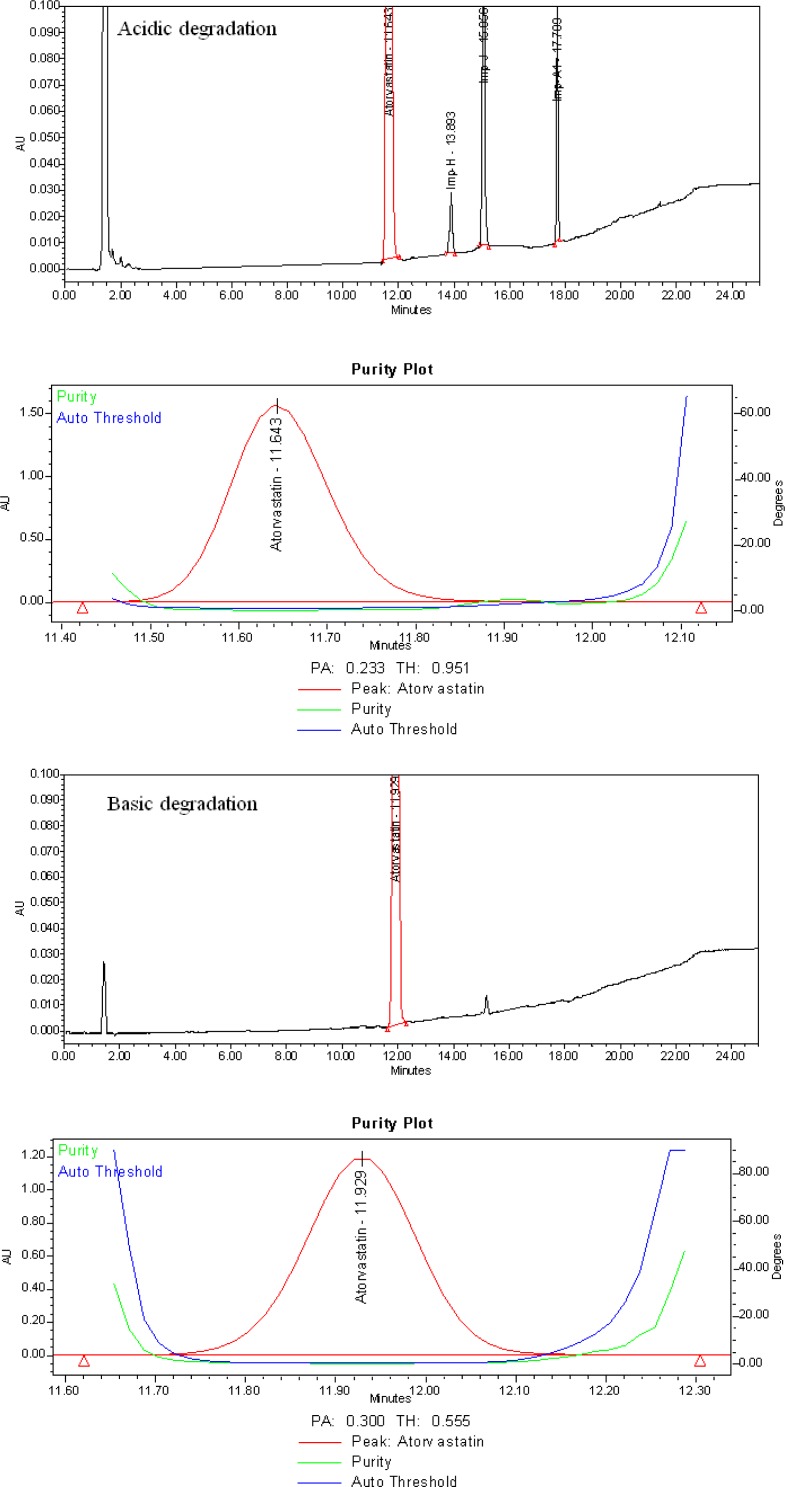

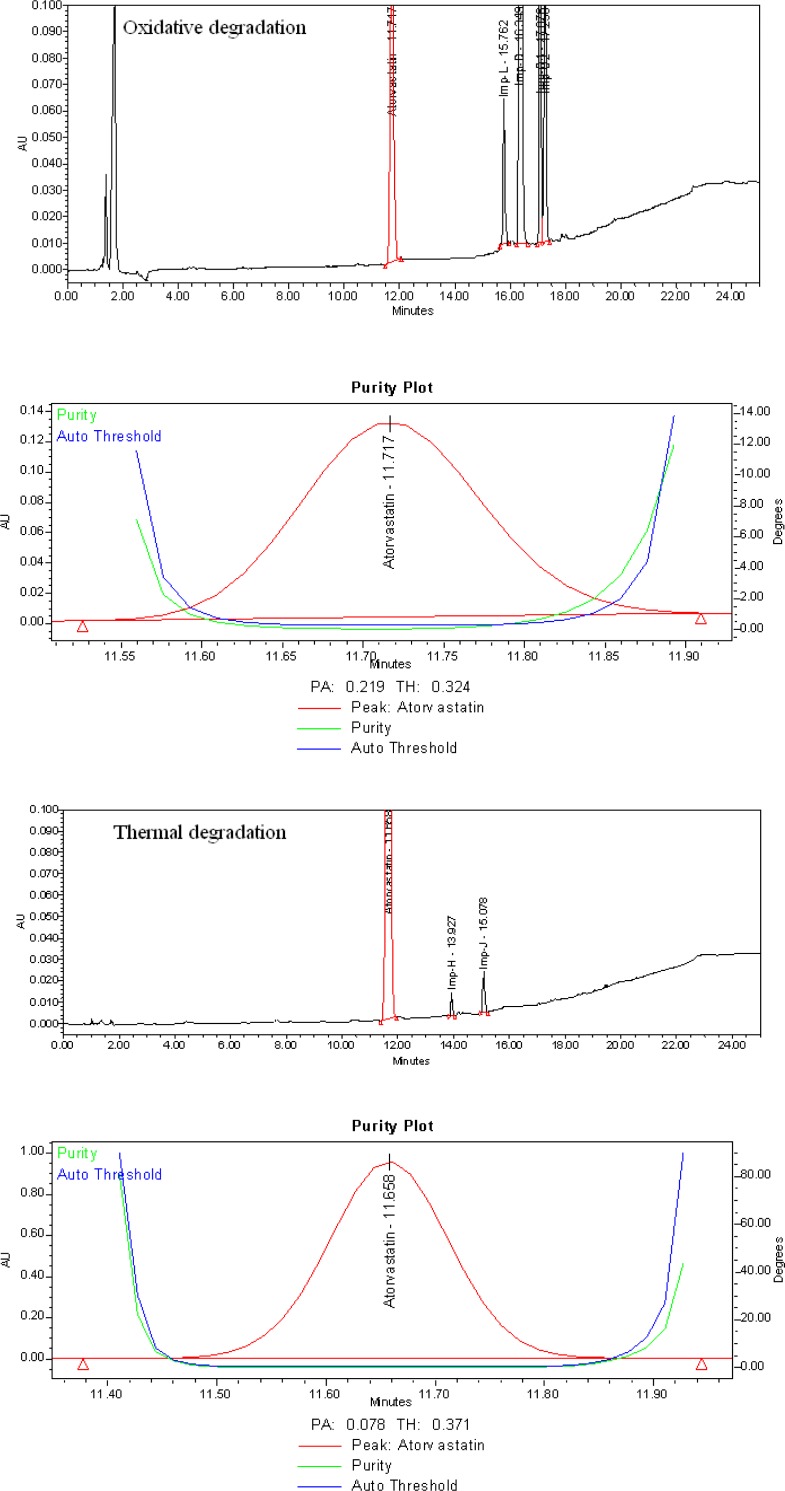

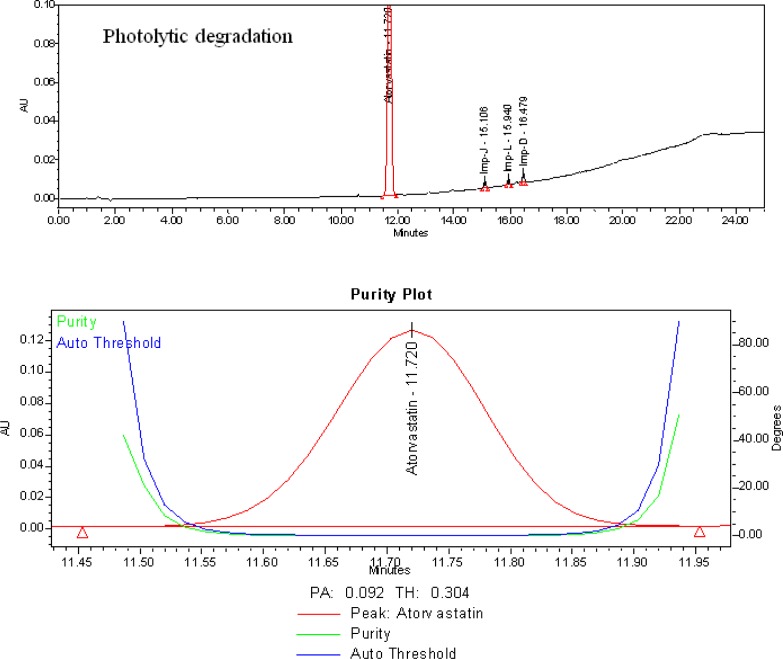
Specificity in presence of degradation products

The degradation of the drug substance was observed in most of the stressed conditions like acidic hydrolysis, oxidative, thermal, and photolytic conditions. Degradation was not observed when the analyte was subjected to base hydrolysis. The chromatograms are presented in Fig. 4. The drug substance Atorvastatin calcium under acidic hydrolysis led to the formation of unknown degradation of impurity A1 along with the known impurities H & J, under oxidative stress conditions led to the formation of unknown degradation impurities O1 & O2 along with the known impurities L & D, under thermal stress conditions led to the formation of known impurities H & J, and under photolytic stress conditions led to the formation of known impurities J, L, and D. The peak purity index over the single point threshold obtained in all stressed samples for the analyte peak demonstrated the specificity. Also, the peak homogeneity study by PDA/LCMS confirmed that the Atorvastatin calcium peak was homogeneous and pure in all of the analyzed stress samples. The assay of Atorvastatin calcium was unaffected in the presence of its potential impurities, and its degradation products confirmed the specificity and stability-indicating ability of the developed method. The mass balance was 99.5% w/w when the RRF of the degradant was considered to be one. All of the studies confirmed the specificity and stability-indicating ability of the developed LC method. The summary of the forced degradation is captured in Table 3 and Table 4.

### Identification of major degradants formed in acidic and oxidative degradations

A LC–MS study was carried out to determine the m/z value of the major degradation products formed during acidic hydrolysis and oxidative degradation using an Agilent 1100 series liquid chromatography system coupled with a 6410 series triple quadruple mass spectrometer. The LC-MS conditions are already described in Section 2.4. The m/z values obtained for the unknown degradation products Imp-A1, Imp-O1, and Imp-O2 were 523.6, 591.6, and 591.6, respectively and corresponds to molecular weights 522.6, 590.6, and 590.6, respectively. The proposed structures for degradation impurities are shown in Fig. 1.

### Precision

The precision of an analytical procedure expresses the closeness of agreement among a series of measurements obtained from multiple samplings of the same homogenous sample under prescribed conditions. The RSD for the assay results of Atorvastatin calcium during the method precision study was less than 0.2% and the RSD for the content of all 12 impurities were within 2.0%. The RSD for the assay results obtained in the intermediate precision study was 0.4 % and the RSD for the content of all 12 impurities were less than 1.5%. Also, the individual values fall well in the range of the confidence interval of average, confirming good precision of the method. The %RSD values are reported in Table 5 & Table 6 for related impurities and Atorvastatin calcium respectively.

### Limit of Detection (LOD) and Limit of Quantitation (LOQ)

**Fig. 5:**
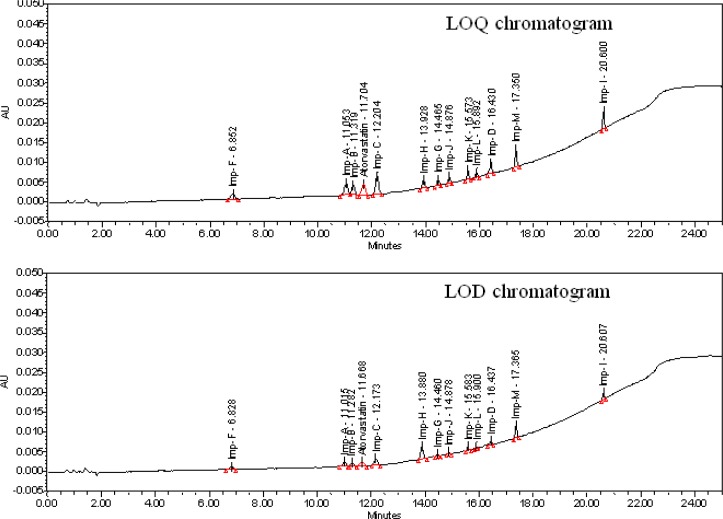
Typical LOQ and LOD chromatograms of Atorvastatin calcium with its related impurities

The LOD of Atorvastatin calcium and its 12 potential impurities were less than or equal to 0.02% w/w (of TAC) for a 10 μL injection volume (Fig. 5). The LOQ of Atorvastatin calcium and its 12 potential impurities were less than or equal to 0.05% w/w (Fig. 5). Since the dosage of Atorvastatin calcium is between 10 to 80 mg per day, the LOQ at the reporting threshold for the known impurities and the API holds good for the necessity of the method. These limits of quantification levels of the impurities were helpful for the process research work to control the impurities at the accepted level during the optimization of the process. The RSD of impurity content at the LOQ level was less than 2.0 % and the recovery values at the LOQ level were from 98.1 to 102.1. The LOD & LOQ values of Atorvastatin calcium and its related impurities, and the precision at the LOQ level are tabulated in Table 5. The results of the accuracy at the LOQ level are tabulated in Table 7.

### Linearity

The linear calibration plot for the assay was obtained over the calibration ranges tested, i.e. 400–600 μg/mL and the correlation coefficient obtained was greater than 0.999. The values of slope, intercept, and %Y-intercept of the calibration curves were determined. An excellent correlation existed between the peak area and concentration of Atorvastatin calcium for the assay determination. The results are computed in Table 6.

The linear calibration plot for the related substances was obtained over the calibration ranges tested, i.e. the LOQ-0.30% w/w for Atorvastatin calcium and its 12 potential impurities. The correlation coefficients obtained for all impurities were also greater than 0.999. The values of slope, intercept, and %Y-intercept of the calibration curves were determined. The RRF of each impurity was determined using the slope of each impurity plot against the Atorvastatin calcium plot. The Y-intercept of each plot was within the 2.0% of the response at 0.15% w/w level of each impurity, describing that the plot goes almost through the origin. This enables the obtainment of an exact value of RRF which will minimize the error in the quantification of impurities. Linearity results and RRF values are computed in Table 5.

### Accuracy

The percentage recovery of all 12 impurities in bulk drug samples ranged from 98.5 to 103.1 (Table 7). The percentage recovery of Atorvastatin calcium in bulk drug samples ranged from 99.5 to 99.9 (Table 6). All of the individual recovery values of the assay and impurities were well within the confidence interval of mean values. Good recovery values were obtained, reflecting the exact values of RRF for all impurities as well as the capability of method accuracy.

### Robustness

In all of the deliberate varied chromatographic conditions carried out as described above (flow rate, wave length, temperature, and organic modifier), the tailing factor of Atorvastatin calcium was less than 1.1, the theoretical plates were more than 60,000, and the resolution between Atorvastatin, Imp-B, and Imp-C was greater than 1.5. A very minor variation in the theoretical plates, resolution, and tailing factor results was observed in all of the robustness conditions, thus illustrating the robustness of the method. Though the higher column temperature showed a better resolution, it is preferable to run in a nominal temperature when considering the durability of the column. The results are shown in Table 8.

### Solution stability and mobile phase stability

During solution stability experiments, it was observed that the solution was stable for 4 h at room temperature and for 24 h when stored at 5±2 °C. The RSD of the assay of Atorvastatin calcium during the mobile phase stability experiments was within 0.60 %. No significant changes were experienced in the content of any of the impurities during the mobile phase stability experiments. The accuracy of the assay at each time point against the initial value is between 99.6 and 99.9%. The accuracy of the content of each impurity against the initial value is between 97.1 and 102.4%. The mobile phase stability experimental data confirms that the mobile phase used was stable up to 48 h. It is an advantage that from the same run, the assay results and impurity quantifications can be derived. This helps to reduce the analysis time and number of samples that can be analyzed until 48 h in the same sequence in the quality control during the regular analysis.

## Conclusion

The developed, rapid LC method for the related substances and assay determination of Atorvastatin calcium is linear, precise, accurate, and specific. The method was validated following the requirements of ICH and the results were satisfactory. The developed stability-indicating LC method can be used for the routine analysis of production samples and also to check the stability of bulk samples of Atorvastatin calcium during its storage.

Communication number IPDO IPM-00341 has been allotted for this research article in the research laboratory.

## Figures and Tables

**Tab. 1.** Results of system suitability test

**Cpd.**	**RT (min)**	**RRT^a^ (n=3)^c^**	**USP resolution R_s_^b^ (n=3)^c^**	**USP tailing factor T (n=3)^c^**	**No. of theoretical plates (*N* tangent method)**
Imp-F	7.0	0.59±0.01	–	1.0±0.05	42,302
Imp-A	11.2	0.94±0.01	18.4±0.32	1.0±0.02	57,012
Imp-B	11.5	0.97±0.01	2.0±0.41	1.0±0.03	62,354
Atorvastatin	11.9	1.0	2.1±0.57	1.0±0.02	65,869
Imp-C	12.4	1.04±0.01	2.8±0.18	1.0±0.05	67,568
Imp-H	14.0	1.18±0.01	9.1±0.49	1.0±0.01	137,049
Imp-G	14.6	1.23±0.01	3.5±0.67	1.0±0.05	179,352
Imp-J	15.0	1.26±0.01	3.1±0.75	1.0±0.09	201,754
Imp-K	15.6	1.32±0.01	5.5±0.92	1.0±0.02	260,023
Imp-L	16.0	1.34±0.01	2.6±0.61	1.0±0.05	222,493
Imp-D	16.5	1.39±0.01	4.1±0.18	1.0±0.01	226,010
Imp-M	17.4	1.47±0.01	7.2±0.10	1.0±0.01	404,130
Imp-I	20.6	1.74±0.03	29.8±0.12	1.0±0.01	651,954

a Relative retention times (RRT) were calculated against the retention time (RT) of Atorvastatin;

b Resolution calculated between two adjacent peaks;

c Mean ± SD.

**Tab. 2.** Results of method development

**Trial**	**Column**	**Dimensions**	**Mobile phase**	**Conclusion**
1	Zorbax SB-C18	150 X 4.6 mm 5.0 μ	Phosphate buffer with pH 2.5/Acetonitrile	Imp-B and Imp-C were co-eluted with analyte
2	Zorbax XDB-C18	150 X 4.6 mm 3.5 μ	Water/Acetonitrile/Trifluoroacetic acid	Imp-B was co-eluted with analyte
3	Zorbax XDB-C8	150 X 4.6 mm 3.5 μ	Water/Acetonitrile/Trifluoroacetic acid	Imp-B was co-eluted with analyte
4	Zorbax SB-Cyano	150 X 4.6 mm 3.5 μ	Water/Acetonitrile/Trifluoroacetic acid	Imp-B and Imp-C were co-eluted with analyte
5	Zorbax SB-Phenyl	150 X 4.6 mm 3.5 μ	Water/Acetonitrile/Trifluoroacetic acid	Imp-B was co-eluted with analyte

**Tab. 3.** Summary of forced degradation results

**Stress condition**	**Time**	**Purity of**	**Assay of A**		**Remarks**

		**Analyte after degradation**			
Unstressed sample	–	99.8		99.7	–
Acid hydrolysis (0.1N HCl)	24 h	85.3		85.9	Significant degradation was observed. Unknown degradation impurity-A1 along with known impurities-H & J were formed
Base hydrolysis (1N NaOH)	42 h	99.5		99.4	No degradation products formed
Oxidation (1% H_2_O_2_)	24 h	87.1		87.9	Significant degradation was observed. Unknown degradation impurities-O1 & O2 along with known impurities-D & L were formed
Thermal (105°C)	10 d	95.2		94.8	Significant degradation was observed. Known impurities-H & J were formed
Photolytic degradation as per ICH guidelines	11 d	94.7		94.1	Significant degradation was observed. Known impurities-J, L & D were formed

**Tab. 4.** Results of degradation studies

**Degradation product**	**Mass number (m/z)**	**Relative retention time (RR_t_) min**	**Tailing factor (T_f_)**
A1	523.6	1.52	1.0
O1	591.6	1.43	1.0
O2	591.6	1.45	1.0

**Tab. 5.** Results of validation parameters for related impurities

**Parameter**	**Atorvastatin**		**Imp-A**	**Imp-B**	**Imp-C**	**Imp-D**	**Imp-F**

LOD (mg/mL)	0.011		0.013	0.012	0.009	0.010	0.009
LOQ (mg/mL)	0.035		0.044	0.041	0.031	0.034	0.031
Regression equation(y)							
Slope (m)	451.21		472.12	455.91	436.54	604.62	338.4
Intercept (C)	−2.50		−3.20	0.98	2.31	−3.10	0.98
% Y-intercept	−0.21		−0.32	0.12	1.10	−1.10	0.53
Correlation coefficient	0.9999		0.9997	0.9999	0.9992	0.9996	0.9994
Precision at LOQ level (%RSD)^a^	0.98		0.72	1.01	1.24	0.32	1.23
Precision (%RSD)^b^	–		1.10	1.23	1.35	1.24	1.02
Ruggedness(%RSD)^b^	–		0.98	0.93	1.10	1.27	0.98
Relative response factor	1.0		1.0	1.0	1.0	1.30	0.75

**Parameter**	**Imp-G**	**Imp-H**	**Imp-I**	**Imp-J**	**Imp-K**	**Imp-L**	**Imp-M**

LOD (mg/mL)	0.009	0.010	0.011	0.010	0.012	0.010	0.009
LOQ (mg/mL)	0.029	0.034	0.036	0.032	0.041	0.033	0.029
Regression equation(y)							
Slope (m)	466.43	436.15	471.23	432.16	458.94	315.84	453.67
Intercept (C)	−4.7	1.10	−0.98	1.35	1.42	−2.97	−6.12
% Y-intercept	−0.54	0.51	−0.34	1.23	0.24	−0.58	−0.96
Correlation coefficient	0.9999	0.9992	0.9994	0.9998	0.9997	0.9993	0.9995
Precision at LOQ level (%RSD)^a^	1.54	0.21	0.31	0.54	0.68	1.21	1.36
Precision (%RSD)^b^	1.98	0.31	0.45	0.56	0.58	0.60	1.45
Ruggedness(%RSD)^b^	1.21	0.54	0.96	0.63	0.57	0.69	1.21
Relative response factor	1.0	1.0	1.0	1.0	1.0	0.7	1.0

a Linearity range was from LOQ to 0.30 %w/w of Atorvastatin calcium and its related impurities with respect to analyte concentration;

b (n=6).

**Tab. 6.** Results of validation parameters for Atorvastatin calcium at assay level

**Parameter**	**Atorvastatin**
Regression equation (y)	
Slope (m)	234.1
Intercept (C)	−4.2
% Y-intercept	−0.56
Correlation coefficient	0.9999
Precision (%RSD)^b^	0.12
Ruggedness(%RSD)^b^	0.36
% Recovery^c^ at 50% level	99.9 ± 0.13
% Recovery^c^ at 100% level	99.5 ± 0.69
% Recovery^c^ at 150% level	99.7 ± 0.78

a (n=6);

b (n=3).

**Tab. 7.** Evaluation of accuracy for related impurities

**Amount spiked^a^**	**% Recovery^b^**					
	**Imp-A**	**Imp-B**	**Imp-C**	**Imp-D**	**Imp-F**	**Imp-G**

LOQ	101.0 ± 0.32	101.0 ± 0.25	98.9 ± 0.12	98.1 ± 0.56	98.3 ± 0.12	99.9 ± 0.49
80%	99.7 ± 0.69	101.4 ± 0.56	101.3 ± 0.07	100.7 ± 0.17	98.5 ± 0.45	98.6 ± 0.87
100%	102.1 ± 0.82	100.8 ± 0.32	101.1 ± 0.17	100.9 ± 0.23	100.1 ± 0.45	100.9 ± 0.98
120%	100.6 ± 0.24	100.1 ± 0.82	100.6 ± 0.48	100.0 ± 0.32	99.1 ± 0.53	101.1 ± 0.79

**Amount spiked^a^**	**% Recovery^b^**					
	**Imp-H**	**Imp-I**	**Imp-J**	**Imp-K**	**Imp-L**	**Imp-M**

LOQ	98.2 ± 0.87	102.1 ± 0.94	100.8 ± 0.56	101.2 ± 0.48	100.1 ± 0.28	100.3 ± 0.18
80%	100.2 ± 0.52	101.0 ± 0.90	100.7 ± 0.40	101.7 ± 0.23	100.0 ± 0.83	103.1 ± 0.17
100%	101.1 ± 0.46	100.2 ± 0.74	101.1 ± 0.69	100.7 ± 0.68	102.1 ± 0.99	100.9 ± 0.16
120%	98.8 ± 0.89	99.7 ± 0.69	98.5 ± 0.98	100.6 ± 0.23	99.5 ± 0.32	100.6 ± 0.42

a Amount of impurities spiked with respect to specification level;

b (n=3).

**Tab. 8.** Results of robustness parameter

**Actual value**	**Changed value**	**No. of theoretical plates (*N* tangent method)**	**USP tailing factor (*T*)**	**USP resolution (Rs) between between Atorvastatin and**	
				**Imp-B**	**Imp-C**
1.0 mL/min	0.8 mL/min 1.2 mL/min	62,154 82,263	1.1 1.0	2.2 1.6	2.9 2.4
245 nm	243 nm 247 nm	68,968 69,794	1.0 1.0	2.1 2.1	2.8 2.8
40°C	35°C 45°C	61,459 75,936	1.0 1.0	2.0 2.4	2.5 3.1
^a^0/40, 10/50, 15/70, 20/90, 25/90	^a^0/36, 10/45, 15/63, 20/81, 25/81 ^a^0/44, 10/55, 15/77, 20/99, 25/99	65,265 84,789	1.0 1.0	2.2 1.6	2.9 2.5

a (T/%B).
